# Opioid-free total intravenous anesthesia for thyroid and parathyroid surgery: Protocol for a randomized, double-blind, controlled trial

**DOI:** 10.3389/fmed.2022.939098

**Published:** 2022-08-30

**Authors:** Dan Wang, Yu-qin Long, Yan Sun, Ya-juan Zhu, Xiao-mei Feng, Hong Liu, Fu-hai Ji, Ke Peng

**Affiliations:** ^1^Department of Anesthesiology, First Affiliated Hospital of Soochow University, Suzhou, China; ^2^Institute of Anesthesiology, Soochow University, Suzhou, China; ^3^Department of Anesthesiology, University of Utah Health, Salt Lake City, UT, United States; ^4^Department of Anesthesiology and Pain Medicine, University of California, Sacramento, Sacramento, CA, United States

**Keywords:** opioid-free anesthesia, total intravenous anesthesia, postoperative nausea and vomiting, postoperative outcomes, thyroid and parathyroid surgery

## Abstract

**Background:**

Opioid-free anesthesia (OFA) may improve postoperative outcomes by reducing opioid-related adverse effects. This study aims to evaluate the effects of OFA on postoperative nausea and vomiting (PONV), postoperative pain, and 30-day outcomes after thyroid and parathyroid surgery.

**Methods:**

This two-center, randomized, double-blind, controlled trial will include 400 adult patients scheduled for thyroid and parathyroid surgery. Patients will be randomly assigned, 1:1 and stratified by sex and site, to an OFA group (esketamine, lidocaine, and dexmedetomidine) or a control group (opioid-based anesthesia with sufentanil). All patients will receive propofol-based total intravenous anesthesia and PONV prophylaxis with dexamethasone and ondansetron. The primary outcome is the incidence of PONV (defined as experiencing any event of nausea, retching, or vomiting) during the first 48 h postoperatively. The secondary outcomes include the severity of PONV, antiemetic rescue therapy, pain scores at rest and while coughing, need for rescue analgesia, perioperative adverse effects related to anesthetics or analgesics (hypotension, bradycardia, hypertension, tachycardia, desaturation, dizziness, headache, hallucination, and nightmare), time to extubation, length of post-anesthesia care unit stay, length of postoperative hospital stay, patient satisfaction, and a composite of 30-day major adverse events (myocardial infarction, cardiac arrest, cerebrovascular accident, coma, acute renal failure, pulmonary embolism, sepsis, septic shock, deep neck space infection, reintubation, reoperation, blood transfusion, failure to wean off ventilator, and death). Analyses will be performed in the modified intention-to-treat population.

**Discussion:**

We hypothesize that our OFA regimen reduces PONV after thyroid and parathyroid surgery. We will also investigate whether OFA leads to improvements in postoperative pain and major adverse events. Our results will offer evidence for optimizing anesthesia regimens in patients who undergo thyroid and parathyroid surgical procedures.

**Clinical trial registration:**

http://www.chictr.org.cn, identifier: ChiCTR2200059656.

## Introduction

Postoperative nausea and vomiting (PONV) are common complications after anesthesia and surgery (1, 2). Patients after thyroid and parathyroid surgery are at high risk of PONV, with an incidence of 40–60% (3–5). PONV leads to discomfort and unsatisfaction of patients. Moreover, it may increase the risk of postoperative bleeding and airway obstruction, prolong the length of hospital stay, and increase healthcare costs (6–9). Despite the application of prophylaxis, reducing PONV is still a major challenge in anesthesia clinical practice.

Total intravenous anesthesia (TIVA) with propofol reduces the risk of PONV, and perioperative use of opioids increases the risk of PONV (10, 11). Opioid-free anesthesia (OFA) is a multimodal anesthesia strategy using α-2 agonists, N-methyl-D-aspartate (NMDA) antagonists, and local anesthetics to replace opioids (12, 13). Some studies found that OFA was associated with reduced incidence and severity of PONV and decreased postoperative opioid requirements (14–16). However, recent randomized studies questioned the benefits of OFA, suggesting that OFA did not improve PONV or pain outcomes but may even cause harms (17, 18). To date, the definitions of OFA vary significantly in the existing literature, and the effects of OFA on postoperative outcomes are largely unknown.

Recent studies have reported the use of opioid-free analgesia after thyroid and parathyroid surgery. A retrospective chart review of 515 patients undergoing thyroid or parathyroid surgery suggested that postoperative opioid prescription was reduced after the implementation of an opioid-free analgesia protocol (19). A survey study of 90 patients showed that an opioid-free postoperative analgesic regimen was feasible and the patients reported satisfaction with pain control (20). However, those studies are limited by the study design, the definition of opioid free (for postoperative analgesia other than for anesthesia during surgery), and lack of adequate control. No randomized clinical trial has assessed the use of OFA in thyroid and parathyroid surgery.

In this study, we aim to determine the effects of the OFA regimen with esketamine, lidocaine, dexmedetomidine, and propofol vs. the traditional opioid-based anesthesia (OA) regimen with sufentanil and propofol on PONV after thyroid and parathyroid surgery. In addition, we will compare postoperative pain and 30-day major adverse events between the two anesthesia groups.

## Methods

### Ethics and registration

The trial protocol was approved by the Ethics Committee of the First Affiliated Hospital of Soochow University (Approval No. 2022-043) on April 28, 2022. This trial was registered at the Chinese Clinical Trial Registry (http://www.chictr.org.cn, identifier: ChiCTR2200059656) on May 5, 2022. This study will be conducted in accordance with the Declaration of Helsinki. Written informed consent will be obtained from all patients. This protocol follows the guidelines of Standard Protocol Items: Recommendations for Interventional Trials (SPIRIT) statement (21).

### Study design and status

This is an investigator-initiated, two-center, double-blind, parallel-group, randomized controlled trial with superiority design. This study will include a total of 400 adult patients undergoing thyroid and parathyroid surgery at two academic medical centers (Shizi Medical Center and Pingjiang Medical Center) of the First Affiliated Hospital of Soochow University, Suzhou, China. In these two centers, ~2,000 thyroid and parathyroid procedures are performed each year.

**Figure 1:**
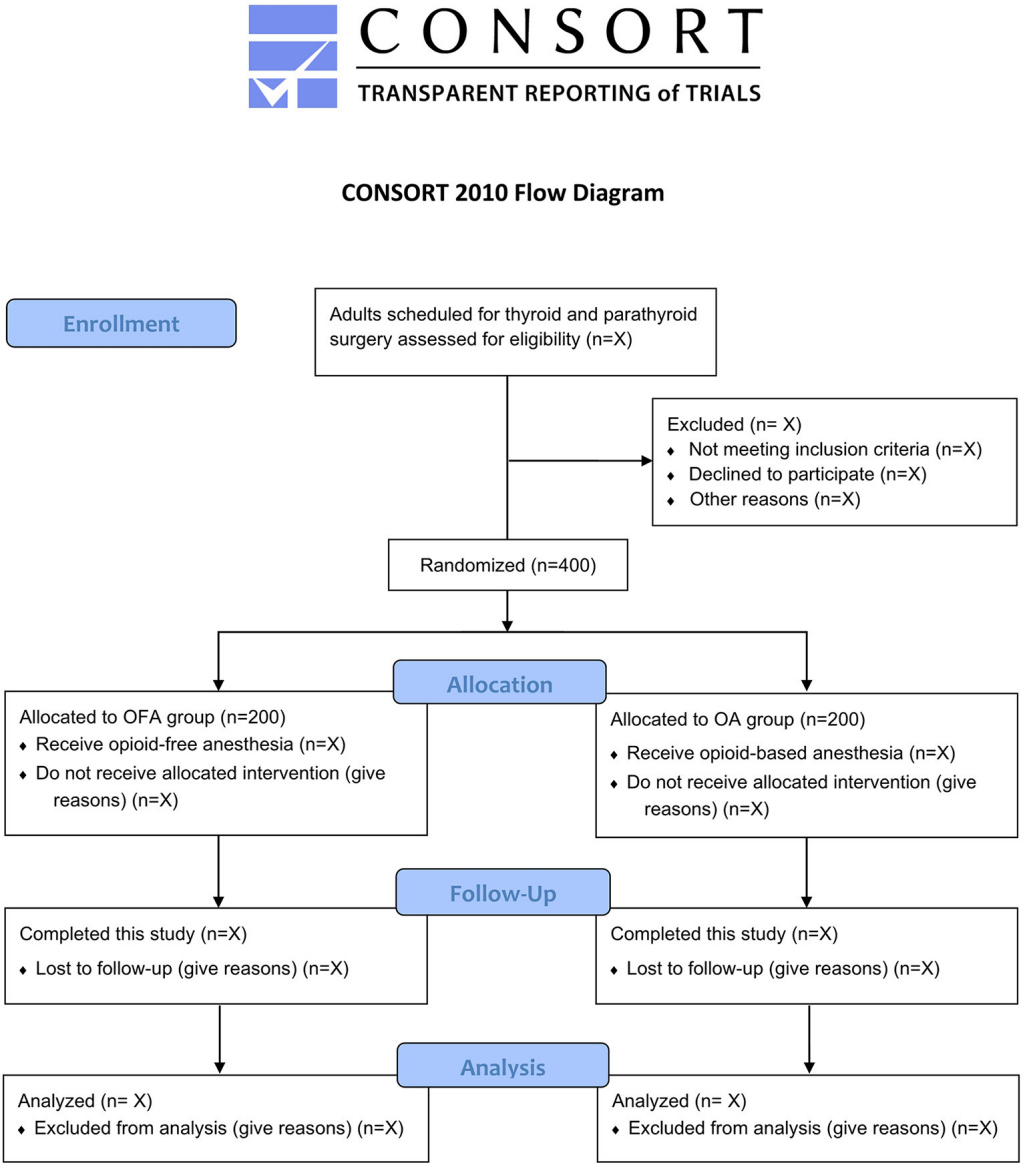
Study flow diagram.

By the time of this manuscript submission, the recruitment of participants has not started. Figure 1 shows the study flow diagram. Table 1 presents the schedule of patient enrollment, study interventions, and outcome measurements in accordance with the SPIRIT statement.

**Table 1:** Schedule of patient enrollment, study interventions, and outcome measurements.

**Timepoint**	**Study period**							
	**Enrollment**	**Allocation**	**Post-allocation**					**Close-out**
	**Preanesthetic visit**	**2 h before surgery**	**During surgery**	**PACU**	**24 h after surgery**	**48 h after surgery**	**Hospital discharge**	**30 day after surgery**
**Enrollment**			
Eligibility screening	×							
Written informed consent	×							
Baseline characteristics	×							
PONV risk score	×							
CESRI score	×							
Randomization		×						
Allocation		×						
**Interventions**								
Opioid-free anesthesia			×					
Opioid-based anesthesia			×					
**Measurements**								
Incidence of PONV				×	×	×		
Severity of PONV				×	×	×		
Antiemetic rescue therapy				×	×	×		
Pain scores at rest				×	×	×		
Pain scores while coughing				×	×	×		
Rescue analgesic use				×	×	×		
Adverse effects^a^			×	×	×	×		
Time to extubation				×				
Length of PACU stay				×				
Postoperative hospital stay							×	
Patient satisfaction						×		
30-day major adverse events^b^								×

According to SPIRIT statement of defining standard protocol items for clinical trials.

PONV, postoperative nausea and vomiting; CESRI, Cervical Endocrine Surgery Risk Index; PACU, post-anesthesia care unit.

^a^Including hypotension, bradycardia, desaturation, dizziness, headache, hallucination, and nightmare.

^b^Including myocardial infarction, cardiac arrest, stroke, coma, renal failure, pulmonary embolism, sepsis, deep neck space infection, reintubation, reoperation, blood transfusions, failure to wean off ventilator, and death.

### Eligibility criteria

Patients who are ≥18 years old with American Society of Anesthesiologists (ASA) physical status I–III and scheduled for thyroid and parathyroid surgery under general anesthesia will be eligible for inclusion. The exclusion criteria are (1) a goiter causing tracheal compression or dyspnea; (2) hyperthyroidism or hypothyroidism; (3) obstructive sleep apnea syndrome; (4) left ventricular ejection fraction <40%, second-degree or greater atrioventricular block, sick sinus syndrome, or severe bradycardia [heart rate (HR) < 50 beats/min]; (5) serious liver or renal dysfunction (Child Pugh grade C or need for renal replacement therapy); (6) epilepsy or seizures; (7) chronic pain or preoperative use of sedatives and analgesics; (8) allergies to any medication in this study; (9) pregnancy or breastfeeding; or (10) inability to communicate preoperatively.

### Randomization and blinding

An independent statistician who will not participate in the subsequent study or data management will generate a randomization list using an online tool (https://www.sealedenvelope.com/randomisation/). The randomization will be done with an allocation ratio of 1:1, block sizes of 2 and 4, and stratification by site and sex. The allocation details will be concealed using sealed opaque envelopes which are stored in a locked cabinet. At each study center, an independent researcher responsible for patient enrollment will inform an independent research nurse to prepare the study medications in identical syringes with the labels of medication number and patient number. All study medications (esketamine, lidocaine, dexmedetomidine, sufentanil, and normal saline placebo) are colorless and clear fluids, so it is impossible to distinguish them. Patients, anesthesiologists, surgeons, other healthcare providers, and investigators responsible for data collection and outcome assessment will be blinded to the group allocation until the completion of final analysis.

### Study interventions

The details of study interventions are shown in Table 2. For anesthesia induction, the OFA group will receive esketamine 0.3 mg/kg (labeled as “Study medication 1”), lidocaine 1 mg/kg (labeled as “Study medication 2”), and propofol 1.5–2.0 mg/kg; the OA group will receive sufentanil 0.3 μg/kg (labeled as “Study medication 1”), normal saline placebo (labeled as “Study medication 2”), and propofol 1.5–2.0 mg/kg.

**Table 2:** Details of study interventions.

	**OFA group (*n* = 200)**	**OA group (*n* = 200)**
**Anesthesia induction**	
Propofol	Propofol 1.5–2.0 mg/kg	Propofol 1.5–2.0 mg/kg
Study medication 1	Esketamine 0.3 mg/kg	Sufentanil 0.3 μg/kg
Study medication 2	Lidocaine 1 mg/kg	Normal saline
**Anesthesia maintenance**	
Propofol	Propofol 50–150 μg/kg/min	Propofol 50–150 μg/kg/min
Study medication 3	Dexmedetomidine 0.5 μg/kg + 0.2 μg/kg/h	Normal saline
Study medication 4	Esketamine 0.1 mg/kg boluses	Sufentanil 0.1 μg/kg boluses

OFA, opioid-free anesthesia; OA, opioid-based anesthesia.

For anesthesia maintenance, the OFA group will receive dexmedetomidine infusion 0.5 μg/kg for 10 min + 0.2 μg/kg/h (labeled as “Study medication 3”), TIVA with propofol 50–150 μg/kg/min, and boluses of esketamine 0.1 mg/kg (labeled as “Study medication 4”) when indicated; the OA group will receive normal saline infusion (labeled as “Study medication 3”) at the same rate as dexmedetomidine, TIVA with propofol 50–150 μg/kg/min, and boluses of sufentanil 0.1 μg/kg (labeled as “Study medication 4”) when indicated.

### Anesthetic management

All patients will fast for 6–8 h and receive no premedication. The baseline blood pressure will be obtained in a preoperative waiting area. After entering the operating room, patients will receive a standard monitoring including electrocardiography, non-invasive cuff blood pressure, and pulse oximetry (SpO_2_). Anesthetic depth will be measured using the Bispectral index (BIS, Aspect Medical Systems, Newton, MA). Intraoperative analgesia will be monitored using the surgical pleth index (SPI, GE Healthcare, Helsinki, Finland).

The OFA group and the OA group will receive the above-mentioned anesthesia regimens, respectively (Table 2). Patients will be endotracheally intubated with intravenous cisatracurium 0.2 mg/kg. Mechanical ventilation will be started with a tidal volume of 8–10 ml/kg (predicted body weight), a frequency of 12–18 breaths/mins, and an inspired oxygen fraction of 50%. The end-tidal carbon dioxide will be maintained at 35–45 mmHg. The depth of anesthesia will be titrated to BIS values of 45–55 *via* the manual adjustment of propofol infusion (22, 23). An adequate intraoperative analgesia will be achieved using dexmedetomidine infusion and boluses of esketamine (the OFA group) or boluses of sufentanil (the OA group), based on the patients' clinical signs and SPI targets within 20–50 (24, 25). Patients will be covered by a warming blanket to maintain a nasopharyngeal temperature of 36–37°C. Intravenous fluid repletion will be provided using the Lactated Ringer's solution.

For PONV prophylaxis, all patients will receive intravenous dexamethasone 5 mg after anesthesia induction and ondansetron 4 mg at the end of surgery. Flurbiprofen axetil 50 mg will be given intravenously at approximately 30 mins before the end of surgery. After tracheal extubation, patients will be transferred to a post-anesthesia care unit (PACU) and receive oxygen supplementation of 3 L/min *via* a nasal catheter. When a modified Aldrete score ≥9 is reached, patients will be discharged from the PACU to the surgical wards (26). For antiemetic rescue therapy, an additional dose of ondansetron 4 mg will be administered. For postoperative pain management, patients will receive intravenous flurbiprofen axetil 50 mg every 12 h during the first two postoperative days. In case of pain scores ≥4 on the numerical rating scale (NRS, ranging from 0 to 10; 0 = no pain and 10 = the most severe pain imaginable), rescue analgesia with intravenous tramadol will be administered.

To improve efficiency and consistency, the anesthetic care and perioperative treatment in each center will be provided by a multidisciplinary team consisting of an attending anesthesiologist and a resident or nurse anesthetist and surgeons who have already performed ≥200 thyroid and parathyroid surgical procedures within the setting of this study. Perioperative care in the two study groups will be identical except for the study interventions.

### Study outcomes

The primary outcome of this study is the incidence of PONV (defined as experiencing any event of nausea, retching, or vomiting) during the first 48 h postoperatively. The secondary outcomes include the severity of PONV, antiemetic rescue therapy, pain scores at rest and while coughing, need for rescue analgesia, perioperative adverse effects related to anesthetics or analgesics (hypotension, bradycardia, hypertension, tachycardia, desaturation, dizziness, headache, hallucination, and nightmare), time to extubation, length of PACU stay, length of postoperative hospital stay, patient satisfaction, and a composite variable of 30-day major adverse events (myocardial infarction, cardiac arrest, cerebrovascular accident, coma, acute renal failure, pulmonary embolism, sepsis, septic shock, deep neck space infection, reintubation, reoperation, blood transfusion, failure to wean off ventilator, and death) (27). The definitions of the major adverse events are based on the National Surgical Quality Improvement Program (NSQIP) from the American College of Surgeons (ACS) (Supplementary Table S1) (28).

The severity of PONV and pain intensity will be assessed at PACU discharge and 24 and 48 h postoperatively. The severity of PONV will be rated using a 4-point Likert scale: 0 = none, 1 = mild (not interfering with activities of daily living), 2 = moderate (sometimes interfering with activities of daily living), and 3 = severe (inability to do any activities of daily living, or ≥3 vomits) (29, 30). Postoperative pain will be measured using the 11-point NRS.

Hypotension (defined as a decrease in mean blood pressure [MBP] > 30% of baseline or MBP < 65 mmHg) will be treated with intravenous ephedrine 6–10 mg or phenylephrine 50–100 μg, and bradycardia (defined as HR < 50 beats/min) will be treated with intravenous atropine 0.3–0.5 mg. Hypertension (defined as an increase in MBP > 30% of baseline) will be treated with intravenous urapidil 5 mg. Tachycardia (defined as HR > 100 beats/min) will be treated with intravenous esmolol 20 mg. Desaturation (defined as SpO_2_ < 95%) after extubation will be treated with oxygen supplementation of 5–10 L/min *via* the nasal catheter. If patients show signs of upper airway obstruction, maneuvers of jaw thrust or chin lift, insertion of an oropharyngeal airway, or ventilation with face mask or laryngeal mask will be applied. The episodes of dizziness, headache, hallucination, and nightmare within 48 h after surgery will be recorded. At 48 h postoperatively, patients will be asked to rate their satisfaction with anesthesia and analgesia on a 5-point Likert scale (5 = highly satisfied, 4 = satisfied, 3 = neutral, 2 = dissatisfied, and 1 = very dissatisfied) (31).

### Data collection and registration

Trained independent investigators who are unaware of group assignment will collect patients' demographic data and baseline characteristics. The proven risk factors of PONV are female, non-smoker, history of motion sickness or PONV, and postoperative use of opioids (10). Based on the number of risk factors, the Apfel's PONV risk score will be calculated (ranging from 0 to 4, with each point indicating that the patient has a 20% increased risk of developing PONV) (10). In addition, patients will be assessed for risk of major postoperative adverse events using the Cervical Endocrine Surgery Risk Index (CESRI) developed from the ACS NSQIP (27). The primary and secondary outcome measures as well as other non-outcome perioperative data will be collected. The values of MAP and HR will be recorded at seven timepoints: baseline, anesthesia induction, immediately after tracheal intubation, skin incision, 30 mins into the surgery, the end of surgery, and immediately after tracheal extubation. Total doses of propofol, esketamine, lidocaine, sufentanil, and dexmedetomidine will be documented. The 30-day postoperative follow-up data will be collected *via* reviewing electronic medical records and telephone calls.

All data will be entered into the electronic case report forms (eCRFs) under the supervision of trained research personnel. The electronic trial database will be established based on the eCRFs. After the completion of data registration, the electronic database will be locked. After de-identification, the database will be sent to an independent statistician from the Department of Epidemiology and Biostatistics, School of Public Health, Medical College of Soochow University for final analyses according to the prespecified statistical plan. The process of data collection and registration will be monitored by the data monitoring committee (DMC) independent of the trial investigators. The DMC consists of three independent clinicians (a professor of clinical anesthesiology, a professor of surgery, and a pharmacologist).

### Safety monitoring

The dose or infusion rate of the study medications in both groups are well within the current clinical norms. Therefore, we anticipate that serious adverse events during this study would be rare. Any adverse event related to the study interventions or not must be reported within 24 h to the DMC on a “Adverse Event Form.” In case of serious adverse events such as unexpected deterioration of patients' clinical status during surgery, the attending anesthesiologists could request unmasking of allocation and adjust or discontinue the administration of study medications. The DMC will conduct an ongoing review to assess the safety and safeguard the interests of patients. Based on the safety data, the DMC will make recommendations to pause or stop this study.

### Sample size calculation

Previous studies and meta-analysis reported that the incidence of PONV after thyroid and parathyroid surgery under opioid-based propofol anesthesia was ~44% (3–5). We expect that the incidence of PONV would be 30% in patients receiving the OFA strategy, that is an absolute reduction of 14%. Based on this assumption, 183 patients in each group would be required with a power of 80% and at an α level of 0.05. To allow for possible dropouts, a total of 400 patients (n = 200 in each group) will be enrolled. The sample size is calculated using the PASS software (version 15.0.5, NCSS, LCC, Kaysville, UT).

### Statistical analysis

The demographic data and baseline characteristics will be presented using descriptive statistics only, without conducting between-group comparisons. Continuous data will be presented as means and standard deviations, or medians and interquartile ranges, depending on data distribution. Categorical data will be presented as numbers and percentages. Between-group differences will be analyzed using the independent t test, repeated measures analysis of variance, or Mann-Whitney rank sum test, Chi-squared test, or Fisher's exact test, as appropriate.

For the primary and secondary outcomes, the therapeutic effects of study interventions will be assessed using mean difference or odds ratio with 95% confidence intervals. The primary outcome of PONV incidence will be further analyzed using a multivariate logistic regression model adjusting for several baseline covariates (sex, smoking status, history of motion sickness or PONV, and trial site). Subgroup analyses for the incidence of PONV will be performed according to sex (female vs. male), Apfel's PONV risk score (0–2 vs. 3–4), and trial site. As for the secondary outcomes, corrections will be made for multiple comparisons by calculating the false discovery rate using the Benjamini–Hochberg method.

The analyses will be carried out based on the modified intention-to-treat population, including all randomized patients undergoing thyroid and parathyroid surgery with the primary outcome measurement available. We have no plans for missing data imputation or interim analysis. All data will be analyzed using the SPSS software (version 19.0; IBM SPSS, Chicago, IL) by the independent statistician. A two-sided *P* value < 0.05 indicates a statistically significant difference.

## Discussion

This two-center, randomized, double-blind, controlled trial will include 400 adults who undergo thyroid and parathyroid surgery. We will assess the effects of OFA vs. OA on the incidence and severity of PONV, rescue antiemetic use, postoperative pain, need for rescue analgesia, adverse effects related to anesthetics or analgesics, time to extubation, length of PACU stay, length of postoperative hospital stay, patient satisfaction, and 30-day major adverse events. Our primary hypothesis is that our OFA strategy using esketamine, lidocaine, dexmedetomidine, and propofol would reduce PONV during the first 48 h postoperatively. Moreover, we will explore whether OFA would decrease postoperative pain and reduce major adverse events. The implementation of this trial and reporting of results will conform to the Consolidated Standards of Reporting Trials guidelines (32).

Opioid medications are the standard treatment for surgical stress response and postoperative pain (33, 34). However, perioperative opioid administration is associated with adverse effects, such as PONV, sedation, respiratory depression, ileus, delirium, and hyperalgesia (35, 36). The increasing opioid use also contributes to opioid addiction, epidemic of opioid abuse, and opioid-related deaths (36–38). Many patients have their first exposure to opioids in the perioperative settings. Moreover, recent clinical studies and laboratory data suggested a possible relationship between the use of opioids and cancer recurrence. A retrospective study reported that intraoperative use of ketorolac was associated with a reduced cancer recurrence rate when compared with other analgesics (sufentanil, ketamine, or clonidine) (39). Opioids have been reported to inhibit cellular and humoral immune function (e.g., morphine inhibits the NK cell cytotoxicity), but the interaction and mechanisms are not fully understood (40). Based on the recently published consensus practice guideline from the Society of Onco-Anesthesia and Perioperative Care, multimodal analgesia consisting of paracetamol, non-steroidal anti-inflammatory drugs, oral pregabalin/gabapentin, with or without dexamethasone should be encouraged starting from the preoperative period in patients undergoing head neck onco-surgeries (Grade of recommendations: A/B; Level of consensus: strong consensus) (41).

In this context, multimodal analgesia and OFA strategies are being investigated to reduce opioid-related adverse effects and associated risks. In our OFA regimen, intraoperative anti-nociception will be provided with the use of esketamine, lidocaine, and dexmedetomidine. Esketamine is an S (+) isomer of ketamine, acting on NMDA receptors to exert anti-nociceptive and anesthetic effects (42). Compared with racemic ketamine, esketamine has the advantages of less adverse effects and a shorter recovery time (43). In addition, esketamine has been shown to counter the remifentanil-induced respiratory depression (44). Lidocaine is a local anesthetic that can be used intravenously for anti-nociception (45). Studies suggested that intravenous lidocaine reduced postoperative pain, opioid consumption, PONV, and length of hospital stay (46–49). The recommended initial dose of intravenous lidocaine is <1.5 mg/kg (50). Dexmedetomidine is an α-2 agonist possessing sedative, sympatholytic, and analgesic effects (51). Our recent studies have shown the favorable effects of perioperative dexmedetomidine for patients undergoing cardiac or aortic surgery (52–54).

Ziemann-Gimmel et al. (16) reported that their opioid-free TIVA strategy with ketamine, dexmedetomidine, and propofol reduced the incidence and severity of PONV after bariatric surgery. However, anesthesia maintenance was different between the two groups (TIVA with propofol vs. inhalational anesthetics), which may have confounded their results. A recent randomized study showed that OFA with dexmedetomidine, esketamine and sevoflurane did not reduce PONV or pain after gynecological laparoscopic surgery (17). A significant limitation is that a higher sevoflurane concentration was used in the OFA group than that in the OA group. Generally, inhalation anesthesia is associated with a higher risk of PONV compared to TIVA with propofol (55, 56). Beloeil et al. (18) investigated the effects of standard balanced anesthesia with either remifentanil plus morphine or dexmedetomidine (the OFA group) on opioid-related adverse events. However, their study was stopped early due to severe bradycardia in five patients and asystole in three of them. Dexmedetomidine was infused at 1.2 ± 2 μg/kg/h until the end of surgery. With such a high dose of dexmedetomidine, it is plausible that the associated risks of OFA outweigh the benefits.

Until now, OFA has been used for several types of surgery (such as bariatric, breast, gynecological, spinal, and coronary artery bypass grafting) (57–59), but its effect in thyroid and parathyroid surgery has yet to be determined. In our OFA regimen, we will use a low dose of dexmedetomidine infusion (0.2 μg/kg/h), intravenous lidocaine (1 mg/kg), and esketamine (a single dose of 0.3 mg/kg during induction and additional boluses of 0.1 mg/kg intraoperatively) for intraoperative analgesia. To reduce PONV, all our patients will receive propofol-based TIVA and PONV prophylaxis with dexamethasone and ondansetron. Thus, the first strength of our study is that it represents the first randomized clinical trial to assess the effects of OFA on PONV, postoperative pain, and 30-day outcomes after thyroid and parathyroid surgery. Another strength is that we plan to enroll a total of 400 patients from two medical centers in this trial, which is an adequate number of patients powered for the primary outcome.

Our study has several limitations, first, the optimal approach to administer OFA during thyroid and parathyroid surgery remains unknown. We believe that the choice and doses of medications (including esketamine, lidocaine, and dexmedetomidine) in our OFA regimen are not definitive, and other ways to administer OFA need to be explored. Second, although the sample size is not that small, this study may still be underpowered for the secondary endpoints. Last, this trial will be conducted at two centers of a university hospital in eastern China, and the generalizability of our results to other medical institutions warrants further investigation.

In conclusion, this randomized clinical trial is designed to determine the effects of OFA, as compare to OA, on PONV in adults undergoing thyroid and parathyroid surgery. We expect that our OFA regimen would lead to decreased incidence and severity of PONV as well as improvements in postoperative pain and major adverse events in these patients.

## Data availability statement

The original contributions presented in the study are included in the article/Supplementary material, further inquiries can be directed to the corresponding author.

## Ethics statement

The studies involving human participants were reviewed and approved by Ethics Committee of the First Affiliated Hospital of Soochow University. The patients/participants provided their written informed consent to participate in this study.

## Author contributions

All authors listed have made a substantial, direct, and intellectual contribution to the work and approved it for publication.

## Funding

This study will be supported by Beijing Health Alliance Charitable Foundation (S033) and Science and Technology Development Plan Clinical Trial Project (SLT201909).

## Conflict of interest

The authors declare that the research was conducted in the absence of any commercial or financial relationships that could be construed as a potential conflict of interest.

## Publisher's note

All claims expressed in this article are solely those of the authors and do not necessarily represent those of their affiliated organizations, or those of the publisher, the editors and the reviewers. Any product that may be evaluated in this article, or claim that may be made by its manufacturer, is not guaranteed or endorsed by the publisher.
