# Supplementary material for: Opioid-free total intravenous anesthesia for thyroid and parathyroid surgery: Protocol for a randomized, double-blind, controlled trial
Source: Front Med (Lausanne). 2022 Aug 30;9:939098. doi: 10.3389/fmed.2022.939098 (PMC9468489; doi:10.3389/fmed.2022.939098)
Supplement: Supplementary file 1 [file Table_1.DOCX]

**Table S1. Definitions of major adverse events within 30 days after surgery according to the ACS NSQIP**

| **Event** | **Definition** |
| --- | --- |
| Myocardial infarction | An acute myocardial infarction which occurs intraoperatively or within 30 days following surgery as manifested by one of the following: 1. Documentation of ECG changes indicative of acute MI (one or more of the following): -ST elevation >1 mm in two or more contiguous leads; -New left bundle branch; -New q-wave in two of more contiguous leads. 2. New elevation in troponin greater than 3 times upper level of the reference range in the setting of suspected myocardial ischemia. |
| Cardiac arrest | The absence of cardiac rhythm or presence of chaotic cardiac rhythm, intraoperatively or within 30 days following surgery, which results in a cardiac arrest requiring the initiation of CPR, which includes chest compressions. Patients are included who are in a pulseless VT or Vfib in which defibrillation is performed and PEA arrests requiring chest compressions. Patients with AICD that fire but the patient has no loss of consciousness are excluded. |
| Cerebrovascular accident | Patient develops an embolic, thrombotic, or hemorrhagic vascular accident or stroke with motor, sensory, or cognitive dysfunction (e.g., hemiplegia, hemiparesis, aphasia, sensory deficit, impaired memory) that persists for 24 or more hours. If a specific time frame for the dysfunction is not documented in the medical record, but there is a diagnosis of a stroke, assign the occurrence, unless documentation specifically states that the motor, sensory, or cognitive dysfunction resolved. |
| Coma | Patient is unconscious, or postures to painful stimuli, or is unresponsive to all stimuli (exclude transient disorientation or psychosis) for >24 hours. Drug-induced coma (e.g., Propofol drips) are excluded. |
| Acute renal failure | A patient without dialysis preoperatively has worsening renal dysfunction postoperatively requiring hemodialysis, peritoneal dialysis, hemofiltration, hemodiafiltration, or ultrafiltration. Placement of a dialysis catheter is indicative of the need for dialysis, if used within 48 hours of placement. |
| Pulmonary embolism | Lodging of a blood clot in the pulmonary artery with subsequent obstruction of blood supply to the lung parenchyma. The identification of a new blood clot in a pulmonary artery causing obstruction (complete or partial) of the blood supply to the lungs. A pulmonary embolism must be noted within 30 days after the principal operative procedure AND the following criteria, A AND B below: A. New diagnosis of a new blood clot in a pulmonary artery AND B. The patient has a V-Q scan interpreted as high probability of pulmonary embolism or a positive CT exam, TEE, pulmonary arteriogram, CT angiogram, or any other definitive imaging modality (including direct pathology examination such as autopsy. |
| Sepsis | Sepsis is the systemic response to infection. Report this variable if the patient has two of the following clinical signs and symptoms of SIRS: -Temp >38 ℃ or <36 ℃; -Heart rate >90 bpm; -Respiratory rate >20 breaths/min or PaCO_2_ <32 mmHg; -While blood cells >12000 cell/mm^3^, <4000 cells/mm^3^, or >10% immature (band) forms; -Anion gap acidosis defined by either: [Na + K] – [Cl + HCO_3_ (or serum CO_2_)] >16 or Na – [Cl + HCO_3_ (or serum CO_2_)] >12. And either A or B below: A. One of the following: -positive blood culture; -clinical documentation of purulence or positive culture from any site for which there is documentation noting the site as the acute cause of sepsis. B. The patient must meet SIRS criteria within 48 hours after the Principal Operative Procedure AND One of the following findings during the Principal Operative Procedure: -Confirmed infarcted bowel requiring resection; -Purulence in the operative site; -Enteric contents in the operative site, or -Positive intra-operative cultures. |
| Septic shock | Sepsis is considered severe when it is associated with organ and/or circulatory dysfunction. Report this variable if the patient has sepsis AND documented organ and/or circulatory dysfunction. Examples of organ dysfunction include: oliguria, acute alteration in mental status, acute respiratory distress. Examples of circulatory dysfunction include: hypotension, requirement of inotropic or vasopressor agents. Septic Shock is assigned when it appears to be related to Sepsis and not a Cardiogenic or Hypovolemic etiology. |
| Deep neck space infection | An infection that occurs within 30 days after the operation and the infection appears to be related to the operation and infection involved deep soft tissues (e.g., fascial and muscle layers) of the incision and at least one of the following: -Purulent drainage from the deep incision but not from the organ/space component of the surgical site; -A deep incision spontaneously dehisces or is deliberately opened by a surgeon when the patient has at least one of the following signs or symptoms: fever (>38 ℃), localized pain, or tenderness, unless site is culture-negative; -An abscess or other evidence of infection involving the deep incision is found on direct examination, during reoperation, or by histopathologic or radiologic examination; -Diagnosis of a deep space infection by a surgeon or attending physician. |
| Reintubation | Patient requires placement of an endotracheal tube or other similar breathing tube (Laryngeal Mask Airway, nasotracheal tube, etc) and ventilator support intraoperatively or within 30 days following surgery which is not intended or planned. |
| Reoperation | Patient has an unplanned return to the operating room for a surgical procedure related to either the index or concurrent procedure performed. This return must be within the 30-day postoperative period. The return to the operating room may occur at any hospital or surgical facility. This definition is not meant to capture patients who go back to the operating room within 30 days for a follow-up procedure based on the pathology results from the index or concurrent procedure. |
| Blood transfusion | At least 1 unit of packed or whole red blood cells given from the surgical start time up to and including 72 hours postoperatively. If the patient receives shed blood, autologous blood, cell saver blood or pleurovac postoperatively, count this blood in terms of equivalent units. For a cell saver, every 500 ml's of fluid will equal 1 unit of packed cells. If there are less than 250 ml of cell saver, round down and report as 0 units. If there are 250 ml or more of cell saver, round up to 1 unit. The blood may be given for any reason. If greater than 200 units, enter 200 units. |
| Failure to wean off ventilator | Total duration of ventilator-assisted respirations during postoperative hospitalization >48 hours. This can occur at any time during the 30-day period postoperatively. This time assessment is cumulative, not necessarily consecutive. Ventilator-assisted respirations can be via endotracheal tube, nasotracheal tube, or tracheostomy tube. |

ACS NSQIP, the National Surgical Quality Improvement Program from the American College of Surgeons; SIRS, Systemic Inflammatory Response Syndrome; VT, ventricular tachycardia; Vfib, ventricular fibrillation; CPR, cardiopulmonary resuscitation; PEA, pulseless electrical activity; AICD, automatic implantable cardioverter defibrillator; TEE, trans-esophageal echocardiography; CT, computed tomography.
